# Two-Photon Fluorescent Probes for Amyloid-β Plaques Imaging In Vivo

**DOI:** 10.3390/molecules28176184

**Published:** 2023-08-22

**Authors:** Yi Chen

**Affiliations:** 1 Key Laboratory of Photochemical Conversion and Optoelectronic Materials, Technical Institute of Physics and Chemistry, Chinese Academy of Sciences, Beijing 100190, China; yichen@mail.ipc.ac.cn; 2 School of Future Technology, University of Chinese Academy of Sciences, Beijing 100190, China

**Keywords:** Aβ plaques, fluorescence probe, two-photon imaging, in vivo

## Abstract

Amyloid-β (Aβ) peptide deposition, hyperphosphorylated tau proteins, reactive astrocytes, high levels of metal ions, and upregulated monoamine oxidases are considered to be the primary pathological markers of Alzheimer’s disease (AD). Among them, Aβ peptide deposition or Aβ plaques, is regarded as the initial factor in the pathogenesis of AD and a critical pathological hallmark in AD. This review highlights recently Aβ-specific fluorescent probes for two-photon imaging of Aβ plaques in vivo. It includes the synthesis and detection mechanism of probes, as well as their application to two-photon imaging of Aβ plaques in vivo.

## 1. Introduction

Alzheimer’s disease (AD), the most common form of dementia that affects more than 35 million people worldwide [1], is characterized by extracellular amyloid plaques in the brain [2,3]. The major components of these plaques are aggregated forms of the amyloid-β peptide (Aβ), derived from the proteolytic cleavage of the amyloid precursor protein (APP) by β-secretase followed by γ-secretase [4]. Aβ contains two predominant isoforms: Aβ 40 and Aβ 42. Aβ 40 accounts for more than 90% of total Aβ, while Aβ 42 is more aggregation-prone than Aβ 40 and is the main composition in the plaques [5]. It is widely accepted that Aβ aggregation from monomers to oligomers, protofibrils, and eventually fibrils plays a pivotal role in the pathogenesis of AD [6,7]; therefore, Aβ plaques have been considered as main biomarkers for AD diagnosis [8,9]. The accurate detection and imaging of Aβ plaques can provide important information for early treatment of AD since Aβ plaques appear as early as ~10 years before the clinical symptoms of AD and go throughout the whole disease process [10,11]. Currently, the available approaches for detection of Aβ plaques in the brain through neuroimaging include positron emission tomography (PET) [12,13,14,15], single photon emission computed tomography (SPECT) [16,17,18], magnetic resonance imaging (MRI) [19,20,21], and optical imaging [22,23,24,25,26].

Recently, fluorescent detection and imaging of Aβ plaques has become a burgeoning technology since it provides high sensitivity, fast data analysis, real-time detection, and high-resolution imaging [27,28,29,30,31,32,33], which are necessary characteristics to achieve the early detection of AD. Fluorescent probe, in particular, near-infrared (NIR) fluorescent probe is favorable for Aβ plaque imaging in vivo due to its deep tissue penetration, low autofluorescence, and minimal photodamage. Up to now, numerous NIR fluorescent probes have been developed to detect and/or visualize Aβ plaques in vitro and in vivo [34,35,36,37,38,39,40,41,42] and many are cited in some excellent reviews [43,44,45,46,47,48]. This review focuses on recent progress in NIR fluorescent probes for two-photon brain imaging of Aβ plaques based on organic small molecules. Probes based on complex and nanoparticles are not involved in the review. Two-photon microscopy (TPM) has emerged as an important tool for imaging of biological tissues due to the following merits. (1) Avoids light scattering which is a serious issue with one-photon confocal microscopy, (2) two-photon excitation only occurs on the focal point; therefore, it can provide a high-resolution imaging, and (3) excitation at NIR wavelengths allows for deeper penetration and minimal photodamage and photobleaching to tissues. These promising features have motivated the search for two-photon fluorescent probes for the detection and imaging of biological analytes in vivo [49,50,51,52,53].

## 2. Design of Aβ Plaque-Specific Fluorescent Probes

To better understand specific biomarkers that reliably correlate with AD, researchers have made great efforts in fluorescence imaging of Aβ plaques. Currently, the general structure of a classical NIR fluorescent probe for imaging Aβ species is a highly environment-sensitive fluorophore with D-π-A or D-π-A-π-D structure (D: electron-donor group; A: electron-acceptor group). The type of structure can significantly affect the optical and biological properties of probes, such as emission wavelength, two-photon absorption cross-sections, quantum yield, and blood−brain barrier permeability [54,55,56]. To date, a great deal of NIR fluorescent probes have been developed for the detection and imaging of Aβ plaques and other biomarkers of AD, including dicyanomethylene acceptor derivatives [57,58,59], difluoroboronate incorporated curcumin scaffold [60,61,62], and BODIPY derivatives [63,64,65]. Many reviews [66,67,68,69,70,71,72,73] have successively reported them.

**Figure 1:**
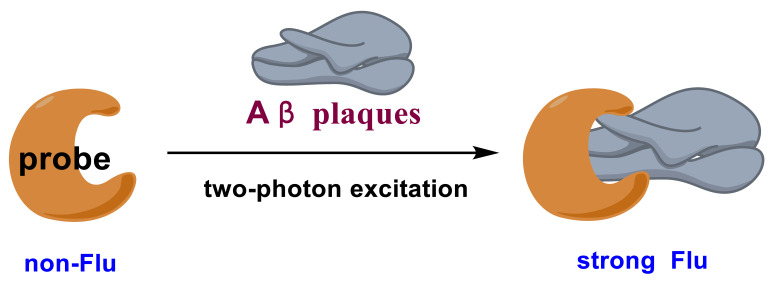
Design strategy of fluorescent probes for Aβ plaques.

This review focuses on the recent development of two-photon fluorescent probes and their application to TPM imaging of Aβ plaques in vivo. A successful candidate should have some merits of specific response to Aβ plaques, excellent two-photon cross-sections (δ_TPA_ or ϕδ_TPA_), NIR fluorescence, and large fluorescence signal-to-background ratios (SBR). To meet the last requirement, fluorescent probes should be designed to have no fluorescence before the interaction with Aβ plaques; upon the interaction with Aβ plaques, a strong NIR fluorescence could be detected (Figure 1).

## 3. Fluorescent Detection and Imaging of Aβ Plaques

**Figure 2:**
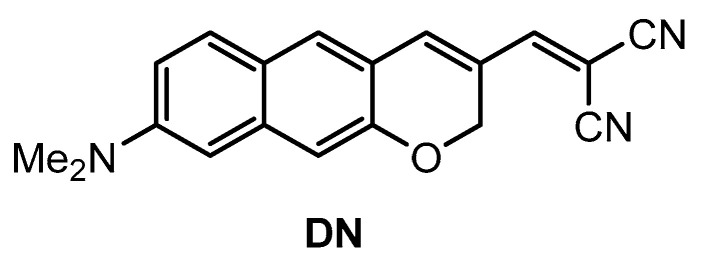
Structure of probe **DN**.

Kim and co-workers [74] developed a π-extended acedan derivative for Aβ plaques detection and two-photon imaging. Acedan, 6-acetyl-2-(dimethylamino)naphthalene, and its derivatives are environmentally sensitive fluorophores and have nonsymmetric D-π-A structures with large two-photon absorption properties, which benefit bioimaging applications [75,76,77]. Probe **DN** (Figure 2) was synthesized from a common intermidiate, 6-(dimethylamino)-3-hydroxy-2-naphthaldehyde. By employing a Baylis−Hillman reaction, introduction of the enone moiety enclosed in the new ring was performed.

**Figure 3:**
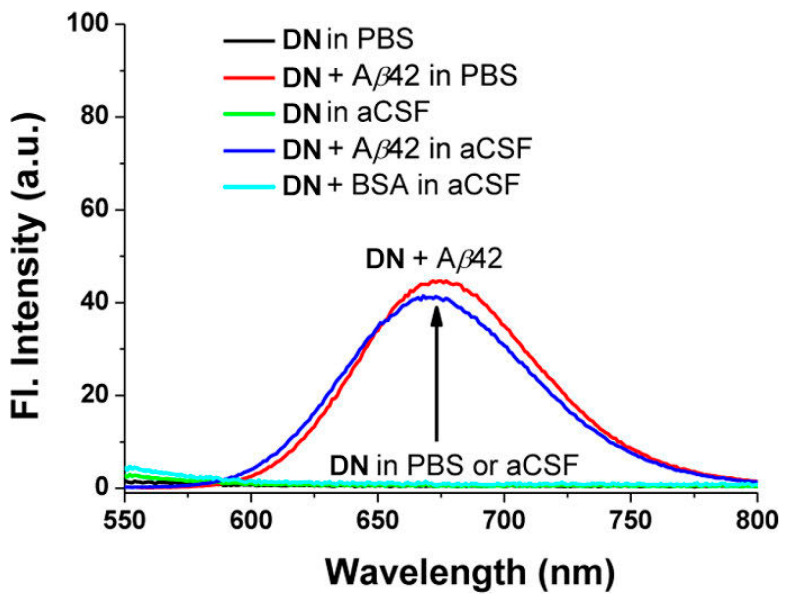
Fluorescence changes of probe **DN** (10 μM) in the presence or absence of Aβ42 aggregates (20 μM) and BSA (20 μg mL^−1^) in PBS buffer (10 mM, pH 7.4, containing 1% DMSO) or in artificial cerebrospinal fluid (aCSF, containing 1% DMSO), measured at 25 °C after mixing for 1 h under excitation at 500 nm. Reproduced with permission from Ref. [74]. Copyright 2015 American Chemical Society.

Probe **DN** exhibited the maximum absorption and fluorescence at 512 nm (λ_abs_) and 679 nm (λ_em_), respectively. In organic solution, a small fluorescence quantum yield (5% in EtOH, 9% in CH_3_CN, 14% in CH_2_Cl_2_) was obtained. The fluorescence was almost quenched when **DN** was in PBS solution (pH 7.4) or in artificial cerebrospinal fluid (aCSF). Upon addition of Aβ42 aggregates into the solution of DN in PBS or aCSF, a large fluorescence enhancement (41- to 60-fold) was obtained (Figure 3). A control experiment demonstrated that bovine serum albumin (BSA) showed negligible interference. The dissociation constant of **DN** with Aβ42 aggregates was determined to be *K*_d_ = 44.6 ± 4.2 nM. The lipophilicity of **DN** was calculated to be log *p* = 3.5, which is close to the optimal value range considered for the blood–brain barrier (BBB) permeation (2.0–3.5) [78].

**Figure 4:**
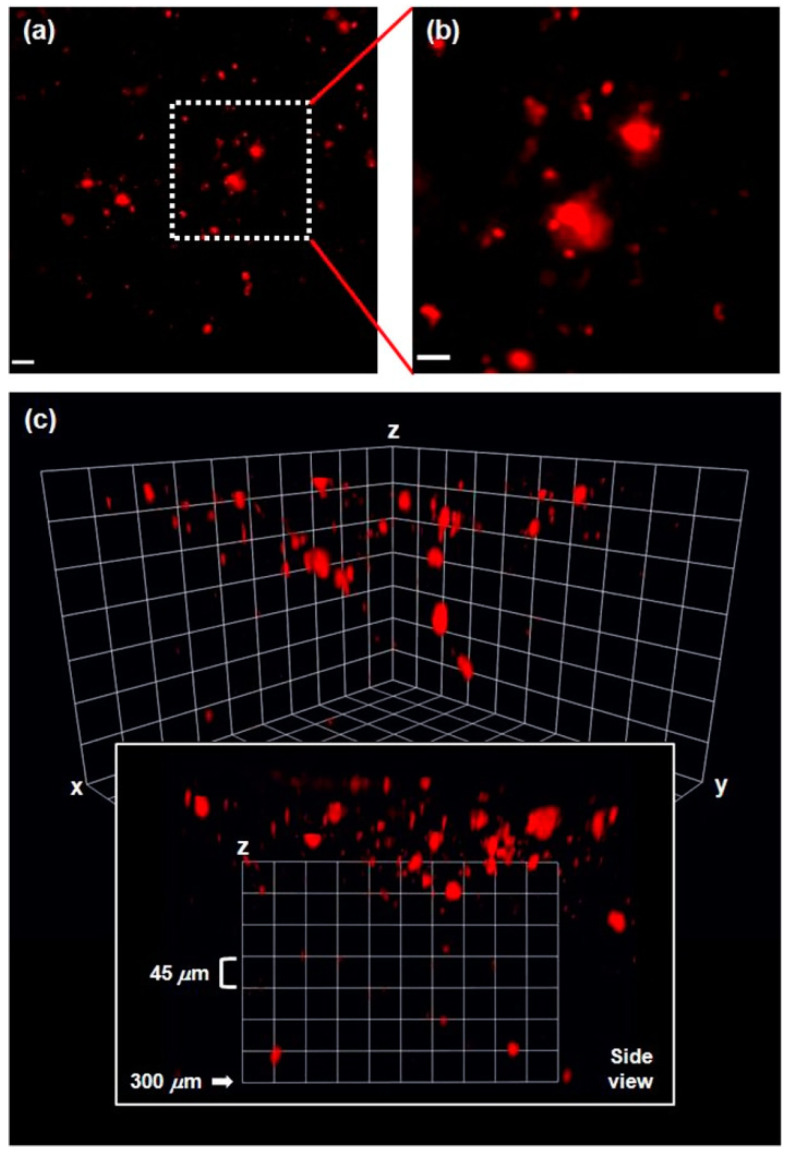
(**a**) In vivo TPM images of Aβ plaques in the frontal cortex of a 5XFAD mouse after ip injection of **DN** (10 mg kg^−1^), 20× magnified at the depth of 50 μm (scale bar: 20 μm). (**b**) Magnified images (60×) of the square area in (**a**) (scale bar: 10 μm). (**c**) 3D images: the images were acquired with 20× magnification along the z-direction at the depth of up to 300 μm from the surface of the cortex, under excitation at 1000 nm with approximately 50 mW laser power at the focal point. Reproduced with permission from Ref. [74]. Copyright 2015 American Chemical Society.

In vivo two-photon microscopy (TPM) imaging of Aβ plaques with probe **DN** was carried out in a live AD mouse model. **DN** was intraperitoneally injected into the 5XFAD mouse (10 mg kg^−1^, one time injection), and fluorescence imaging of the brain was conducted under two-photon excitation at 1000 nm. Clear and bright red fluorescence images of Aβ plaques were obtained (Figure 4), which also confirmed that **DN** readily penetrated BBB. In addition, the 3D images obtained down to 300 μm depth showed that Aβ plaques were spreading out to the cortex region [79] (Figure 4c side view). Furthermore, co-staining experiments showed well-merged fluorescence images by using MeO-X04, a known Aβ plaque-staining fluorescent probe [80], confirming that **DN** efficiently images Aβ plaques.

**Figure 5:**
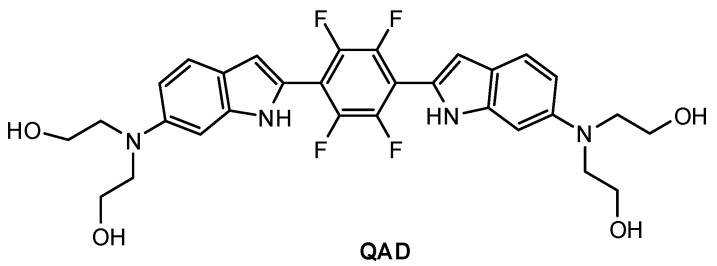
Structure of probe **QAD**.

Mook-Jung and co-workers [81] designed a quadrupolar fluorescent probe for detection and imaging of Aβ42 plaques. Quadrupoles with D–π-A–π-D structure have been regarded as a promising motif for large δ_TPA_ values [82]. Probe **QAD** (Figure 5) was synthesized by coupling reaction of 4-dialkylamino-2-nitrobenzaldehyde and bisphosphonate-substituted tetra-fluorobenzene first, followed by reduction-induced cyclization. **QAD** exhibited almost no fluorescence in PBS buffer (pH 7.4) in the absence of Aβ42 aggregates. Upon addition of Aβ42 aggregates into the solution of **QAD** in PBS (pH 7.4), the fluorescence of **QAD** at 546 nm increased dramatically. The dissociation constant (*K*_d_) of probe **QAD** with Aβ42 aggregates was found to be 16.2 nM. A control experiment demonstrated that BSA and human serum albumin (HSA) showed negligible interference. The lipophilicity value (log P) of **QAD** was calculated to be 3.42 by partitioning between n-octanol and PBS buffer. A large two-photon action cross section (δ_TPA_ = 420 GM) was obtained at 750 nm.

**Figure 6:**
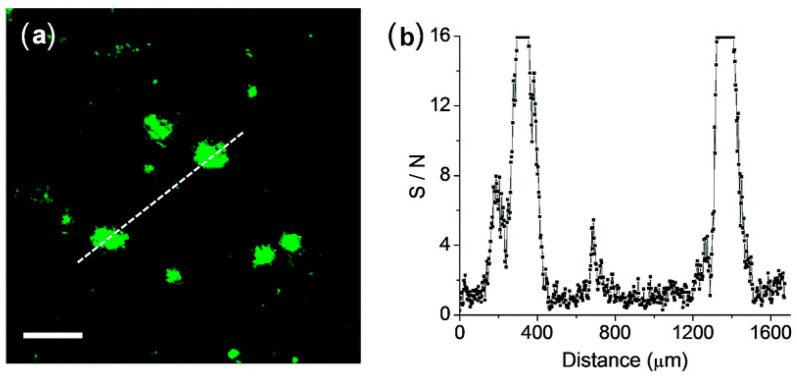
(**a**) TPM images of a cortical slice of brain from transgenic 5XFAD mice stained with 20 mM of probe **QAD** for 90 min and (**b**) Signal-to-noise (S/N) ratio values measured by TPEF intensity of bright cluster and background regions along the white dotted lines in (**a**). The two-photon fluorescence intensities were collected at 450–520 nm upon excitation at 750 nm with fs pulse. Scale bars: 48 mm. Reproduced with permission from Ref. [81]. Copyright 2016 Royal Society Chemistry.

The feasibility of **QAD** for the detection of Aβ plaques in brain tissues was confirmed by TPM images in cortical slices which were taken from a transgenic 5XFAD mouse, an AD model mouse forming Aβ plaques in the brain [83]. Bright spots in TPM imaging were observed in the **QAD**-labeled slice with good S/N ratio (Figure 6).

**Figure 7:**
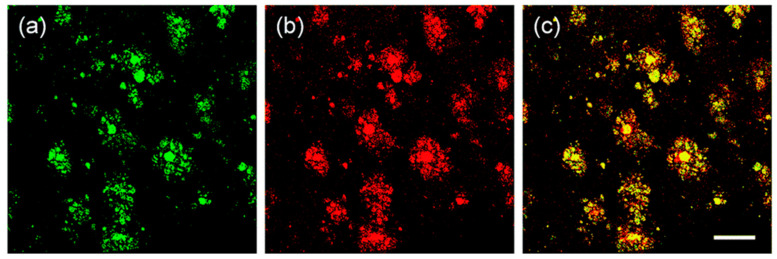
TPM images of cortical slices of brain from transgenic 5XFAD mice co-labeled with (**a**) probe **QAD** and (**b**) Congo red for 90 min, and (**c**) merged image by 20× magnification. Scale bars: 72 µm. Reproduced with permission from Ref. [81]. Copyright 2016 Royal Society Chemistry.

Specific location of **QAD** in Aβ plaques was confirmed by a co-localization experiment between **QAD** and Congo red, a known fluorescent marker for histology of Aβ plaques [84]. The bright fluorescence regions of **QAD** merged well with signals from Congo red with a Pearson’s co-localization coefficient of 0.85 (Figure 7).

**Figure 8:**
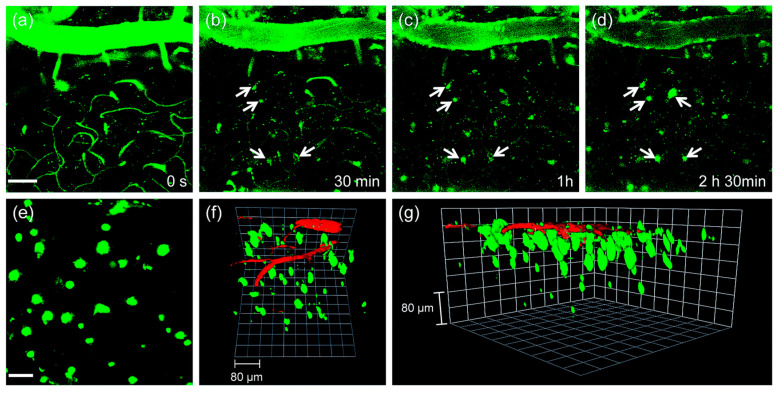
In vivo TPM imaging of the frontal cortex of transgenic 5XFAD mice at (**a**) 0, (**b**) 30, (**c**) 60 and (**d**) 150 min after i.v. injection of probe **QAD** (10 mg kg^−1^). (**e**) 230 sections of images along the z-direction at the depth of ~300 µm from the surface of the cortex were accumulated to visualize Aβ plaque distribution. (**f**,**g**) 3D-reconstructed two-photon image of the frontal cortex of transgenic 5XFAD mice after i.v. injection of probe **QAD** (10 mg kg^−1^) and dextran 40 kDa-Texas red. Approximately 270 sections of images were acquired along the z-direction at a depth of ~300 µm from the surface of the cortex. Scale bars: (**a**) 50 and (**e**) 30 µm. Reproduced with permission from Ref. [81]. Copyright 2016 Royal Society Chemistry.

The utility of probe **QAD** in vivo imaging was evaluated in transgenic 5XFAD mice. As shown in Figure 8, the initial images showed bright fluorescence through the blood vessels in the cortex region (Figure 8a) upon excitation with 780 nm; the bright fluorescence at the vessels decreased with a concomitant increase at the plaques (white arrows in Figure 8b–d) until it reached a peak. Kinetic studies revealed that the circulating half-life (*t*_1/2_) at the vessels was 35.7 min. Both time constants for BBB penetration and for plaque binding were *t*_0_ = 23.4 min and Δτ = 46.9 min, respectively. The 3D images were constructed from approximately 270 sections in which a known blood marker, dextran 40 kDa-Texas red, was injected. Aβ plaques with different sizes at the specific positions were clearly visualized along with blood vessels (Figure 8f,g). In addition, cerebral amyloid angiopathy (CAA), other deposited Aβ aggregates surrounding the wall of blood vessels of the central nervous system [85], were also directly observed.

**Figure 9:**
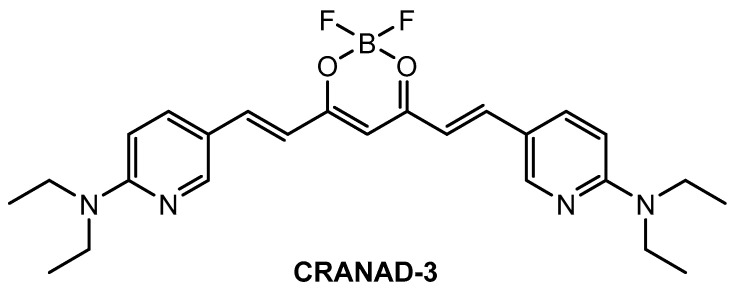
Structure of probe **CRANAD-3**.

Ip and co-workers [86] employed a known fluorophore **CRANAD-3** (Figure 9) as probe for in vivo deep two-photon imaging of Aβ in an AD transgenic mice model. **CRANAD-3** was reported early to be able to image insoluble Aβ aggregates and soluble Aβ monomers and dimers in vitro [87]. The two-photon fluorescence properties of **CRANAD-3** in living brain tissue were characterized in the brain of live APP/PS1 mice with the injections of **CRANAD-3**. It was found that the fluorescence excitation wavelength of **CRANAD-3** is about 900 nm, much longer than that of MeO-X04 (720–750 nm), and the emission peak is about 630 nm, which suggested that **CRANAD-3** is a potentially appropriate probe for two-photon deep brain imaging.

**Figure 10:**
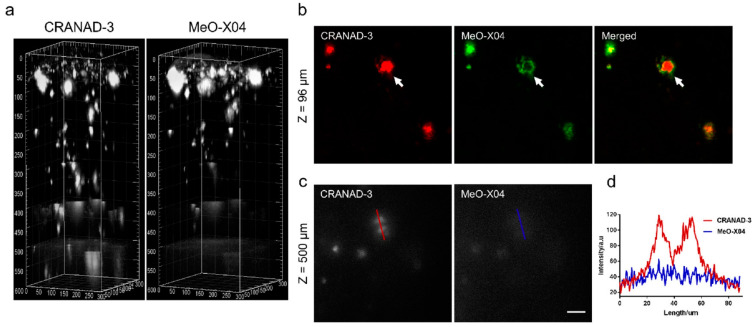
Imaging depth comparison of **CRANAD-3** and MeO-X04. (**a**) Deep brain imaging of amyloid plaques in a 17-month-old APP/PS1 mouse coinjected with **CRANAD-3** (4 mg/kg) and MeO-X04 (5 mg/kg). The z-step in the stack is 2 μm. The 3D image was reconstructed based on the z-stack TPEF images using the commercial Imaris software. (**b**) TPEF images at upper layer (96 μm depth) in (**a**), showing differences in plaque labeling of **CRANAD-3** and MeO-X04, a dense-core plaque is indicted by the white arrow. (**c**) TPEF images at deeper region (500 μm depth) and (**d**) quantitative comparison of SBR of amyloid plaques in (**c**). Scale bar: 40 μm. Reproduced with permission from Ref. [86]. Copyright 2018 American Chemical Society.

In vivo imaging of Aβ plaques with **CRANAD-3** was evaluated in APP/PS1 mice by comparing with MeO-X04. **CRANAD-3** and MeO-X04 were co-injected into the mice with dosages and delivery routes following individual protocols. Both **CRANDA-3**- and MeO-X04-labeled Aβ plaques of the cortex were imaged with excitation wavelengths of 900 and 760 nm, respectively. The well-co-localized images (Figure 10b) confirmed that **CRANDA-3** could specifically image Aβ plaques. Furthermore, **CRANDA-3** showed better labeling efficiency than MeO-X04. The image contrast of **CRANDA-3** surpassed MeO-X04 significantly at deep cortical layers especially in the depth beyond 500 μm (Figure 10c), and a large SBR (4.1) was obtained (Figure 10d). In addition, the authors also indicated that the maximum depth where Aβ plaques could be detected is 900 µm (SBR~1.0) with **CRANAD-3**.

**Figure 11:**
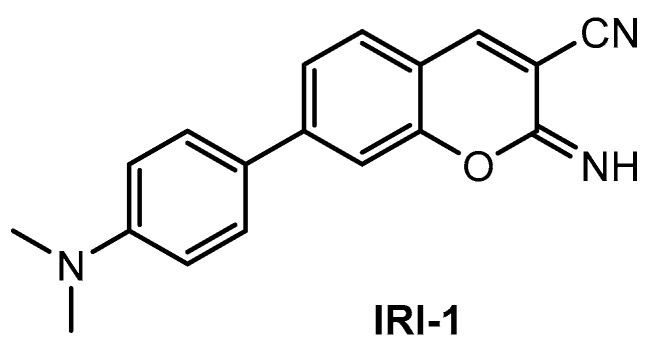
Structure of probe **IRI-1**.

Kim and co-workers [88] described a new two-photon fluorescent probe for detection and imaging of Aβ42 plaques. Probe **IRI-1** (Figure 11) was synthesized by Suzuki coupling reaction between 4-bromosalicylaldehyde and 4-(dimethylamino) phenylboronic acid, followed by condensation and cyclization reaction with malononitrile.

**Figure 12:**
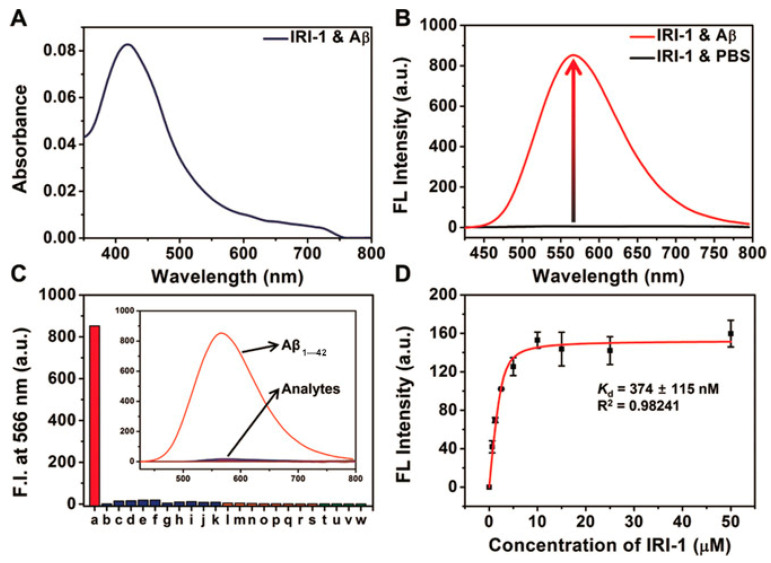
Absorbance and fluorescence data of probe **IRI-1** (10 µm). (**A**) Absorption spectra of **IRI-1** in the presence of Aβ aggregates (20 µm). (**B**) Fluorescence spectra of **IRI-1** in PBS and Aβ aggregates (20 µm). (**C**) Fluorescence response assays (λ_em_ = 566 nm) for **IRI-1** and various potential interferents: a: Aβ aggregates (20 µm), b–k: metal ions (20 µm, from b to k: Al^3+^, Fe^3+^, Fe^2+^, Ca^2+^, Cu^2+^, Zn^2+^, Ni^2+^, Mg^2+^, Na^+^, K^+^), l–s: amino acids (20 µm, from l to s: Lys, Arg, Asp, Glu, His, Trp, Tyr, Phe), and t–w: thiols (20 µm, form t to w: DTT, Hcy, GSH, Cys), in PBS. (**D**) Saturation binding curve of Aβ aggregates (10 µm) as a function of **IRI-1** (0–50 µm) in PBS; error bars represent SD (*n* = 3). Reproduced with permission from Ref. [88]. Copyright 2019 Wiley-VCH.

Probe **IRI-1** exhibited absorption maximum at ~419 nm (Figure 12A). In the absence of Aβ aggregates, no emission was observed, while in the presence of Aβ aggregates in PBS (pH 7.4), a distinct enhanced fluorescence maximum at ~566 nm was detected (Figure 12B). The enhanced fluorescence resulted from two potential pathways: reduced polarity and conformational restriction at the protein binding site. Docking studies showed that there are two main locations for binding affinities of probe **IRI-1** and Aβ aggregates. One is a tunnel along the aggregate axis, and the other is located on a groove along the aggregate axis; the tunnel-based interaction may be more kinetically stable [89]. In addition, a control experiment demonstrated that metal ions, amino acids, and thiols showed negligible interference (Figure 12C), only a weak fluorescence enhancement in the presence of BSA, HSA, or mouse brain homogenates. The binding affinity of probe **IRI-1** toward Aβ aggregates is calculated to be *K*_d_ = 374 ± 115 nm (Figure 12D), and the δ_TPA_ value of **IRI-1** is 111 GM at 880 nm.

**Figure 13:**
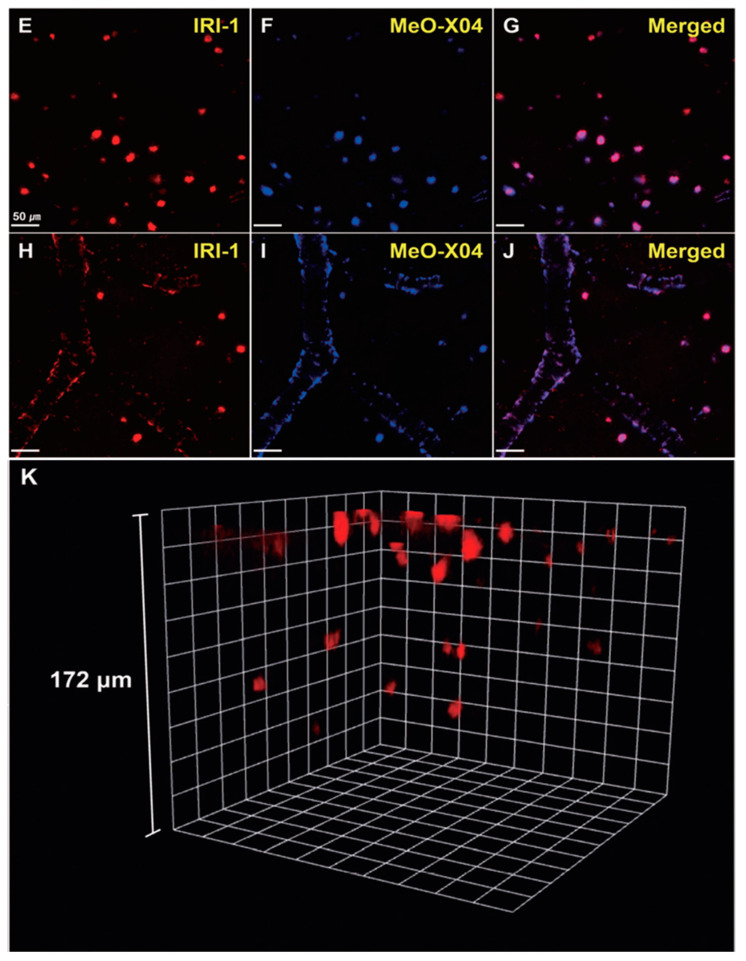
In vivo TPM imaging of Aβ plaques in the frontal cortex of transgenic mice (5xFAD-Tg, 10–12-monthold). Dyes were administered intraperitoneally (5 mg kg^−1^) and imaged with a laser power of around 30 mW at the focal point. (**E**–**J**) Co-staining with **IRI-1** and MeO-X04 of Aβ plaques (**E**–**G**) and CAA near the blood vessel walls (**H**–**J**). Fluorescence images were acquired under excitation at 920 nm (**E**,**H**) and 780 nm (**F**,**I**). Scale bars = 50 mm. (**K**) 3D in vivo imaging of **IRI-1**-stained Aβ plaques. Reproduced with permission from Ref. [88]. Copyright 2019 Wiley-VCH.

In vivo TPM imaging showed that a bright fluorescence in the frontal cortex of 10–12-month old 5XFAD-Tg mice was observed after **IRI-1** was injected into the peritoneal cavity and excitation at 920 nm (Figure 13E). The bright fluorescence is the result of combining lower tissue background emission and large two-photon cross-sections of **IRI-1**. Co-staining experiments (Figure 13E–G) revealed well-merged fluorescence images by using MeO-X04, confirming that **IRI-1** efficiently images Aβ plaques. Moreover, a clear visualization of Aβ deposits on cerebral blood vessels associated with CAA was also found (Figure 13H–J). Finally, 3D TPM imaging showed that individual Aβ plaques could be detected up to a depth of 172 µm (Figure 13K).

**Figure 14:**
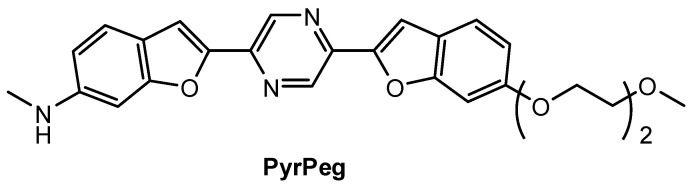
Structure of probe **PyrPeg**.

Lee and co-workers [90] prepared a new NIR fluorescent probe for two-photon imaging of Aβ42 plaques. Probe **PyrPeg** (Figure 14) was synthesized in total of five steps starting from 3-methoxy-N-methylaniline. **PyrPeg** exhibited emission in organic solvents, but almost no emission in PBS solution. A broad two-photon emission in the range of 450–650 nm with a maximum emission at 560 nm was detected when **PyrPeg** was excited with 740 nm excitation in SH-SY5Y cells. Control experiments demonstrated that **PyrPeg** is specific for detection of Aβ42 fibrils over other amyloidogenic proteins. The *K*_d_ values of **PyrPeg** for the binding to the Aβ42 aggregates and monomer were 63.8 and 799 nM, respectively. Such a large difference indicates that the affinity of **PryPeg** for Aβ42 aggregates is much stronger than that for the Aβ42 monomer. Both δ_TPA_ value and logP value of **PyrPeg** are 230 GM (at 740 nm) and 3.51, respectively.

**Figure 15:**
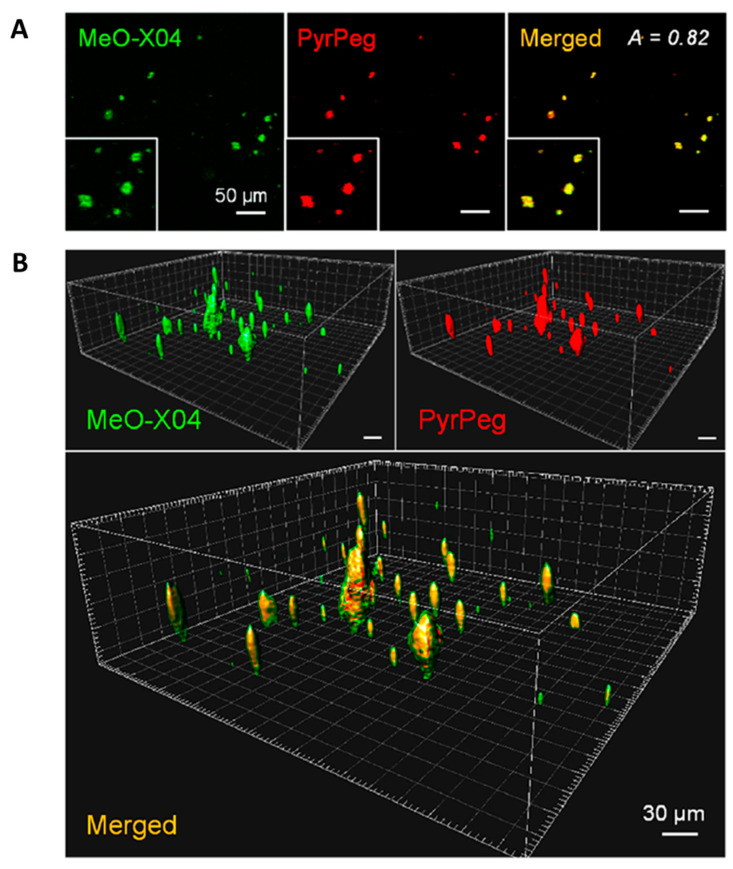
Detection of neuritic plaques in APP/PS1 mice. (**A**) TPM images of an APP/PS1 mouse brain slice 1 day after ip injection with MeO-X04 (2 mg/kg) and iv injection with **PyrPeg** (2 mg/kg) and a merged image (yellow). The images were captured at 400–500 nm (MeO-X04, green) and 530–640 nm (**PyrPeg**, red) upon TP excitation at 750 nm at a depth of 200 μm. (**B**) 3D images constructed from 200 sectional images of the MeO-X04- and **PyrPeg**-injected tissues at a depth of 150–300 μm with 0.75 μm intervals along the z-direction and a merged image. The merged image shows green dots around the overlapping regions. Representative images from replicate experiments (*n* = 3) are presented. Scale bars: 30 and 50 μm. Reproduced with permission from Ref. [90]. Copyright 2020 American Chemical Society.

Co-staining experiments revealed well-merged fluorescence images. As shown in Figure 15, the sectional images captured in Ch1 (detection windows at 400–500 nm for MeO-X04) and Ch2 (detection windows at 530–640 nm for **PyrPeg**) at a depth of 220 μm overlapped well, with the A value of 0.82, which confirmed that probe **PyrPeg** efficiently images Aβ plaques. The 3D images were constructed from 200 sectional images. Both images from Ch1 and Ch2, respectively, overlapped well except for the green dots (MeOX04) scattered around the overlap region (yellow dots) that can be attributed to the tangles and cerebrovascular amyloids [91]. The result indicated that **PyrPeg** was localized in the dense core region of the neuritic Aβ plaque.

**Figure 16:**
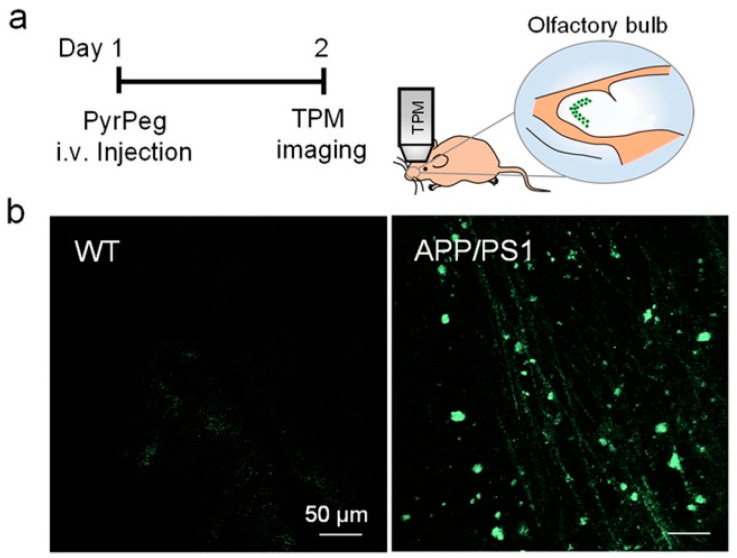
In vivo TPM images of the olfactory bulb in mice. (**a**) Schematic diagram of TPM imaging of the olfactory bulbs of a tail-injected mouse. (**b**) TPM images of the olfactory bulbs of WT (**left**) and APP/PS1 mice (**right**) 1 day after injection with **PyrPeg** (1 mg/kg). Scale bar: 50 μm. Reproduced with permission from Ref. [90]. Copyright 2020 American Chemical Society.

In vivo detection of Aβ plaques was carried out in the olfactory bulb of APP/PS1 mice by TPM imaging. The olfactory bulb is primarily affected in AD [92,93]. Both WT (wide-type) and APP/PS1 mice were injected with **PyrPeg**, respectively, and examined by TPM imaging. As shown in Figure 16, a bright fluorescence was observed in the APP/PS1 mice, but not in the WT mice, indicating that **PyrPeg** can selectively label Aβ plaques in the AD brain, and could be useful for AD diagnosis.

## 4. Conclusions and Outlook

Fluorescence probes can directly detect and image Aβ plaques and other pathological markers in the brain, providing a powerful tool for AD diagnostics in situ during AD development. In this review, recent advances in two-photon Aβ-specific fluorescence probes are highlighted including design strategies and applications to the detection and imaging of Aβ in vivo (Table 1).

To date, a class of two-photon NIR fluorescent probes for Aβ plaques has been developed, but several challenges remain for practical applications. First, sensitivity and selectivity are the basic requirements for accurate detection of AD. Efforts need not only increase the signal-to-noise contrast and improve the sensitivity, but also distinguish diffuse and neuritic plaques and enhance selectivity. Second, for in vivo detection and imaging, NIR or two-photon fluorescent probes should have large two-photon cross-sections and NIR emission as a consequence of their low-power laser excitation (≤5 mW at the focal point does not cause damage to the cells and tissues [94]) and deep penetration depth (>100 µm). Third, appropriate lipophilicity (log P = 2.0–3.5) [79] and good permeability of the blood–brain barrier (BBB) are needed for in vivo detection and imaging in brain tissues. In addition, stability, cytotoxicity, and mechanism are important factors in practical applications which also need to be tested and clarified. With more and more research, outstanding progress of two-photon NIR fluorescent probes for Aβ plaques is expected to be achieved, which will finally improve AD diagnoses and treatments in clinics.

## Figures and Tables

**Table 1:** Highlights of fluorescent probes included in this review for two-photon imaging of Aβ in vivo.

Probe	LogP	λ_ex_ (nm)	δ_TPA_ (GM)	λ_em_ (nm)	Mice	Depth (µm)	Ref
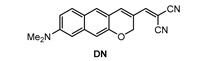	3.5	1000	80	679	5XFAD	300	[74]
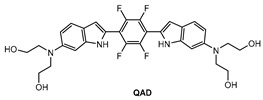	3.4	750	420	508	5XFAD	300	[81]
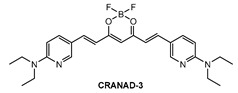	NR	900	NR	630	APP/PS1	500	[86]
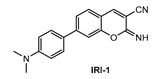	3.3	920	111	566	5XFAD	172	[88]
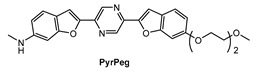	3.5	740	230	560	APP/PS1	220	[90]
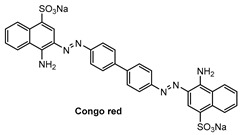							
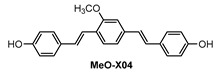							

Probe: fluorescent probe cited in the text for two-photon imaging of Aβ in vivo. Both Congo red and MeO-X04 for co-staining experiments. λ_ex_: two-photon excitation wavelength. δ_TPA_: two-photon action cross section. λ_em_: maximum emission wavelength of probe. Mice: type of mouse. Depth: depth of two-photon fluorescence imaging. NR: no report; Ref: reference.

## Data Availability

Not applicable.
